# A 2/1 Sunitinib Dosing Schedule Provides Superior Antitumor Effectiveness and Less Toxicity Than a 4/2 Schedule for Metastatic Renal Cell Carcinoma: A Systematic Review and Meta-Analysis

**DOI:** 10.3389/fonc.2020.00313

**Published:** 2020-03-06

**Authors:** Huan Deng, Meng Li, Qian Wu, Li Wang, Zhengdong Hong, Fengming Yi, Yiping Wei, Wenxiong Zhang

**Affiliations:** ^1^Department of Thoracic Surgery, The Second Affiliated Hospital of Nanchang University, Nanchang, China; ^2^Department of Urology, The Second Affiliated Hospital of Nanchang University, Nanchang, China; ^3^Jiangxi Medical College, Nanchang University, Nanchang, China; ^4^Department of Oncology, The Second Affiliated Hospital of Nanchang University, Nanchang, China

**Keywords:** renal cell carcinoma, sunitinib, alternative dosing, effectiveness, meta-analysis

## Abstract

**Background:** The standard sunitinib schedule to treat metastatic renal cell carcinoma (mRCC) is 4 weeks on/2 weeks off (4/2). However, some studies revealed intolerable adverse events (AEs) in patients on this schedule. An alternative schedule, 2 weeks on/1 week off (2/1), may overcome this issue. This meta-analysis was performed to compare the effectiveness and toxicity between the 2/1 and 4/2 sunitinib dosing schedules.

**Methods:** We acquired relevant studies by searching PubMed, ScienceDirect, the Cochrane Library, Scopus, Ovid MEDLINE, Embase, Web of Science, and Google Scholar. Our main endpoints included overall survival (OS), progression-free survival (PFS), objective response rate (ORR), disease control rate (DCR), and AEs.

**Results:** We identified 9 medium- and high-quality studies. Both schedules were effective for mRCC, with comparable OS and similar ORR. However, the 2/1 schedule had better PFS (hazard ratio (HR) = 0.81, 95% confidence interval [CI]: 0.66–0.99, *P* = 0.04), higher DCR [risk rate (RR) = 1.22, 95% CI: 1.01–1.47, *P* = 0.04] and fewer dosage interruptions (RR = 0.60, 95% CI: 0.43–0.84, *P* = 0.003). Additionally, the 2/1 schedule elicited fewer specific severe AEs, including thrombocytopenia/platelet disorder, hand-foot syndrome, hypertension, and fatigue. In our subanalysis, PFS was better among East Asians using the 2/1 schedule than among other populations (HR= 0.75, 95% CI: 0.58–0.98, *P* = 0.03), and patients administered an initial dosage of 50 mg/d on the 2/1 schedule had superior PFS (HR = 0.76, 95% CI: 0.59–0.97, *P* = 0.03) than those others.

**Conclusions:** These findings suggest that the 2/1 schedule is more suitable for mRCC than 4/2, due to superior PFS, better DCR and fewer AEs. Nevertheless, more large-scale studies with good quality are needed.

## Introduction

As the second most common tumor in the urological system, kidney cancer is estimated to account for73,820 cancer cases and result in 14,770 deaths in 2019 (1, 2). In addition, over 30% of patients are found to have metastasis at initial diagnosis, and the expenditure for treating metastatic renal cell carcinoma(mRCC) reached nearly $1.6 billion in selected countries (3, 4). The National Comprehensive Cancer Network (NCCN) guidelines have listed sunitinib as a standard first-line antiangiogenic agent to treat mRCC (5).

As a small molecule tyrosine kinase inhibitor (TKI), sunitinib has shown superior efficacy and safety profile among mRCC patients (6). A phase III randomized controlled trial (RCT) indicated that treatment on a 4/2 schedule had an improved PFS, higher response rates, and fewer adverse events (AEs) than interferon-alpha (7). Base on these RCTs, 4 weeks on/2 weeks off (4/2) with a dosage of 50 mg/d is the traditional schedule for sunitinib (8). However, some sunitinib-related severe AEs from the 4/2 schedule led to poor tolerability and reduced health-related quality of life for some mRCC patients, so these AEs need to be monitored carefully (9). This problem requires further research in detail. A new schedule, 2 weeks on/1 week off (2/1), may solve this problem (10). In a phase I study, Britten et al. reported that a 2/1 sunitinib schedule had similar drug accumulation but less toxicity than the 4/2 schedule (11). Although both dosing schedules showed clinical benefits among mRCC patients, the optimal dosing schedule is still controversial. An RCT indicated that the 2/1 schedule had less toxicity with similar progression-free survival (PFS) when compared with the 4/2 schedule (12). However, in a recent study at a major Comprehensive Cancer Center, Atkinson et al. suggested that alternative schedules had superior PFS [hazard ratio (HR) = 0.49, 95% confidence interval [CI]: 0.36–0.67, *P* < 0.0001] and better overall survival (OS) (HR = 0.48, 95% CI: 0.34–0.69, *P* < 0.0001) than traditional schedules (13).

To address this discrepancy, we performed meta-analyses of pertinent articles comparing the antitumor effectiveness and toxicity of the two dosing schedules (2/1 and 4/2) of sunitinib to provide the latest evidence-based suggestions for mRCC.

## Materials and Methods

We conducted the meta-analysis following the PRISMA (Preferred Reporting Items for Systematic Review and Meta-analysis) guidelines (Table S1) (Registration information: PROSPERO CRD42019143043).

### Search Strategies

All pertinent studies were obtained through the following databases: PubMed; ScienceDirect; the Cochrane Library; Scopus; Web of Science; Embase; Ovid MEDLINE; and Google Scholar. We used these terms as follows: “kidney neoplasm,” “sunitinib,” and “alternative dosing schedule.” The complete search strategy in these electronic databases is listed in Table S2. The references of all qualifying studies were searched for potentially eligible articles. Included articles were required to be written in English.

### Selection Criteria

Studies which obeyed these criteria would be enrolled in accordance with PICOS (Participants, Intervention, Control, Outcome, Study design): (1) Participants: patients diagnosed with mRCC (defined as having distant metastasis apart from the primary lesion); (2) Intervention and Control: compared 2/1 schedule vs. 4/2 schedule; (3) Outcome: PFS, OS, objective response rate (ORR), disease control rate (DCR), complete response rate (CR), partial response rate (PR); stable disease rate (SD) and AEs; (4) Study design: RCT or retrospective study (RS); and (5) were written using English.

The reviews without original data, conference abstracts, case reports, meta-analysis, animal experiments, and articles with repeated data would be excluded.

### Data Extraction

The data were independently extracted by two investigators (Deng and Fan) to obtain the following information: first author, publication time, nation, number of participants, participants' features (age, histological types, pretreatment, metastatic sites), antitumor effectiveness index (PFS, OS, ORR, DCR), and AEs (any grade AEs, grade 3–4 AEs). All disagreements were discussed with a third investigator (Zhang) until a consensus was reached. Considering the number and time of events at the same time, we used hazard ratios (HRs) rather than odds ratios to analyze PFS and OS. We obtain HRs and 95% CIs directly from Cox multivariate survival analyses. Otherwise, HRs and 95% CIs were calculated based on Kaplan–Meier curves constructed as indicated by the protocol from Tierney et al. (14).

### Quality Evaluation

The quality of the RCT was appraised by the 5-point Jadad scale including 3 main aspects: randomization, masking, and accountability of all participants. Articles scoring 3 Tierney 5 points were regarded as high-quality (15).

RS' qualities were appraised through the 9-point Newcastle-Ottawa Scale containing these aspects: selection, comparability and exposure. Articles with scores of 8 or 9 were regarded as high quality, while scores of 6 and 7 indicated medium quality (16).

We also made use of GRADE (Grades of Recommendations Assessment, Development and Evaluation) for evaluating therapeutic strategy and the study design regarding the survival, response rates, and toxicity. The GRADE is categorized into4 classes (high, medium, low, and very low) (17).

### Statistical Analysis

We performed this meta-analysis using RevMan (version 5.2) and STATA (version 12.0). HRs with 95% CIs were chosen to analyze PFS and OS (HR > 1 supports 4/2, HR < 1 supports 2/1). We used risk ratios (RRs) with 95% CIs to analyze ORR, DCR (RR > 1 supports 2/1, RR < 1 supports 4/2), and AEs (RR > 1 supports 4/2, RR < 1 supports 2/1). We conducted a subgroup analysis to determine whether the outcomes would be different according to nationality, treatment line, initial dosage, study quality, and study design. We evaluated heterogeneity through the χ^2^ test and *I*^2^ statistic. If *I*^2^> 50% or *P* < 0.10 in the χ^2^ test, showing significant heterogeneity, the random-effects model was applied; otherwise, the fixed-effects model was used. The sensitivity analyses of PFS, OS, ORR, and DCR were performed to strengthen robustness. Publication bias was assessed with Begg's test and Egger's test. *P* < 0.05 showed statistical significance.

## Results

### Search Results and Study Qualities

**Figure 1:**
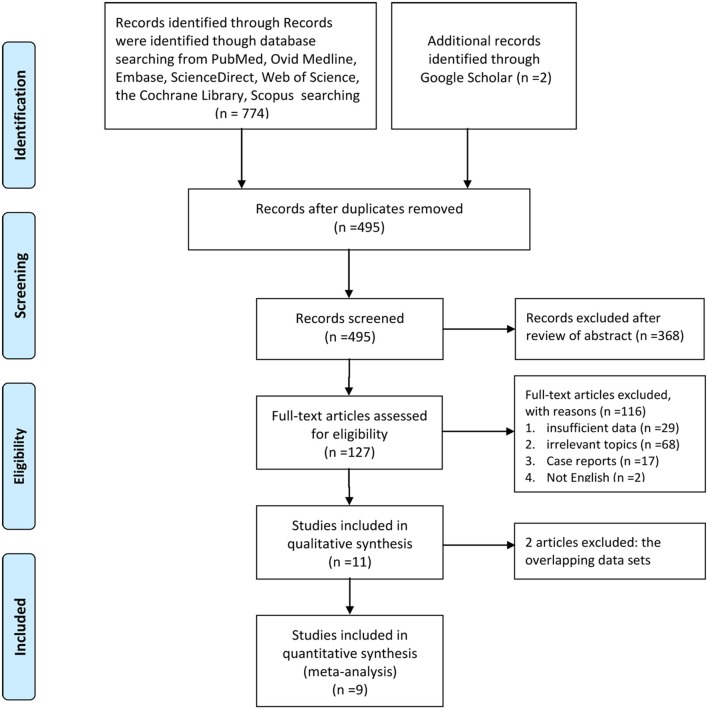
Flow chart of study selection.

Figure 1 illustrates the process of selecting studies. Finally, 9 studies involving 774 patients (2/1 schedule, 264; 4/2 schedule, 510) were selected for this meta-analysis (12, 18–25). One study was an RCT, and the remaining eight studies were RSs. Five articles were considered high quality (1RCT scored four points using the Jadad scale, and4RSs scored eight points using the Newcastle-Ottawa Scale). Four RSs were considered medium quality (three articles scored seven points, and1 article scored six points; Table S3). Furthermore, most of our outcomes were low or very low according to the GRADE scale (Table S4). Table 1 lists the basic features and major assessment indexes of the nine included articles.

**Table 1:** Characteristics of included studies.

**References**	**Country**	**Study period**	**Pre-treatment**	**Groups**	**Treatment line**	**Patients (n)**	**Initial dosage**		**Median age (y)**	**CCRCC (%)**	**Design**	**Score^a^**
							**2/1**	**4/2**				
Lee et al. (12)	Korea	2007.11-2014.02	NPT, CT	2/1 vs. 4/2	NA	38/36	50 mg/d	50 mg/d	57.0/60.0	82/94	RCT	4
Miyake et al. (18)	Japan	2010.01-2017.01	NPT	2/1 vs. 4/2	1	47/62	50 mg/d	50 mg/d	NA	89/87	RS	8
Pan et al. (19)	China	2009.01-2013.07	NPT	2/1 vs. 4/2	NA	32/50	50 mg/d	50 mg/d	66.0/62.0	84/80	RS	8
Ezz El Din et al. (20)	Egypt	2012.01-2016.01	NPT	2/1 vs. 4/2	NA	26/30	50 mg/d	50 mg/d	49.5/49.0	88/77	RS	6
Suo et al. (21)	Canada	2006.01-2012.12	NPT	2/1 vs. 4/2	1,2	9/59	50 mg/d	50 mg/d	62.3/60.8^b^	100/83	RS	7
Knodo et al. (22)	Japan	2010.01-2012.12	NPT	2/1 vs. 4/2	1	26/22	35% pts 50 mg/d	32% pts 50 mg/d	64.6/ 62.7^b^	96/91	RS	7
Zhang et al. (23)	China	2008-2015	NPT	2/1 vs. 4/2	1	24/30	NA	NA	59.5/53.5	62/73	RS	8
Neri et al. (24)	Italy	2008.1-2010.5	NPT	2/1 vs. 4/2	NA	21/10	50 mg/d	50 mg/d	NA	NA	RS	7
Bracarda et al. (25)	Italy	2005.11-2013.8	NA	2/1 vs. 4/2	1	41/211	NA	NA	61.0/59.0	88/87	RS	8

*NPT, nephrectomy; CT, cytokine therapy; 4/2, 4-weeks-on and 2-weeks-off; 2/1, 2-weeks-on and 1-week-off; RCT, randomized controlled trail; RS, retrospective study; pts, patients; CCRCC, clear-cell renal cell carcinoma; NA, not available*.

a *RCT was evaluated using the Jadad scale, and retrospective studies were evaluated using the Newcastle-Ottawa Scale*.

b *Mean*.

### Antitumor Effectiveness

We appraised the antitumor effectiveness between the 2/1 and 4/2 schedules according to PFS, OS, ORR, and DCR.

**Figure 2:**
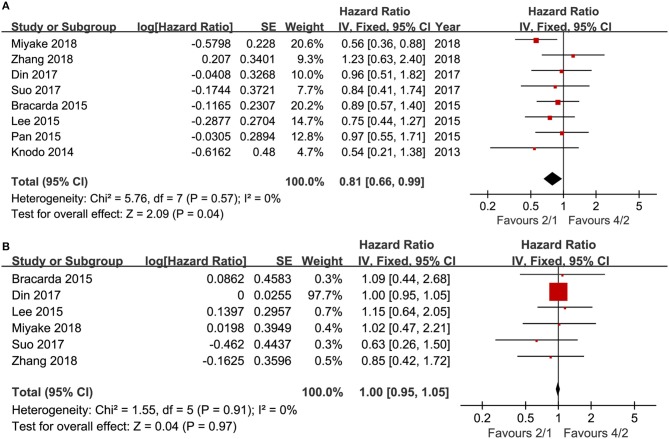
Forest plot of PFS **(A)** and OS **(B)** associated with 2/1 vs. 4/2.

Eight articles compared PFS (heterogeneity: *I*^2^ = 0%, *P* = 0.57). The 2/1 group had an improved PFS compared to that of the 4/2 group (HR = 0.81, 95% CI: 0.66–0.99, *P* = 0.04; Figure 2A).

Six articles compared OS (heterogeneity: *I*^2^ = 0%, *P* = 0.91). No significant differences existed between the two schedules (HR = 1.00, 95% CI: 0.95–1.05, *P* = 0.97; Figure 2B).

**Figure 3:**
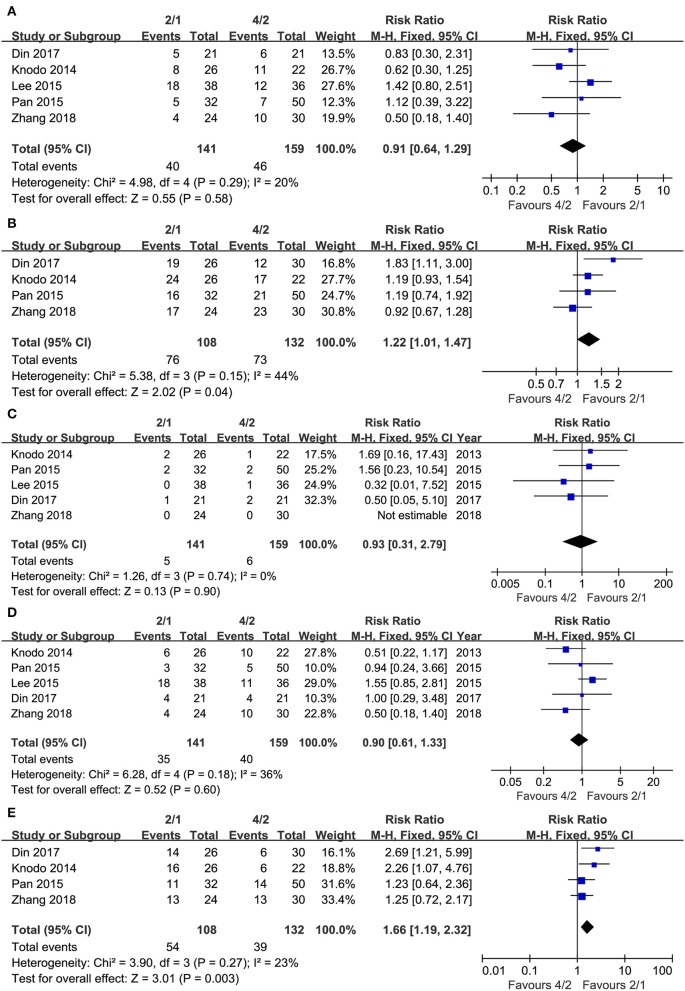
Forest plots of ORR **(A)**, DCR **(B)**, CR **(C)**, PR **(D)**, and SD **(E)** associated with 2/1 vs. 4/2.

Five articles were used to compare ORR (heterogeneity: *I*^2^= 20%, *P* = 0.29). No significant differences existed (RR = 0.91, 95% CI: 0.64–1.29, *P* = 0.58; Figure 3A).

Four articles were used to compare DCR (heterogeneity: *I*^2^ = 44%, *P* = 0.15). The 2/1 schedule had a higher DCR (RR = 1.22, 95% CI: 1.01–1.47, *P* = 0.04; Figure 3B) than the 4/2 schedule.

We also analyzed response rates in detail owing to the contradictory results of ORR as well as DCR. Five articles compared CR (heterogeneity: *I*^2^= 0%, *P* = 0.74). No significant differences existed between the groups (RR = 0.93, 95% CI: 0.31–2.79, *P* = 0.90; Figure 3C). Five studies compared PR (heterogeneity: *I*^2^= 36%, *P* = 0.18). No significant differences existed (RR = 0.90, 95% CI: 0.61–1.33, *P* = 0.60; Figure 3D). Four articles compared SD (heterogeneity: *I*^2^ = 23%, *P* = 0.27), and Figure 3E shows that the 2/1 schedule had more SD (RR = 1.66, 95% CI: 1.19–2.32, *P* = 0.003) than the 4/2 schedule.

### Toxicity

The toxicity of sunitinib between the 2/1 and 4/2 schedules based on any grade as well as on grade 3–4 AEs was compared. In addition, subgroup analyses of the 10 most common toxic events were conducted.

**Figure 4:**
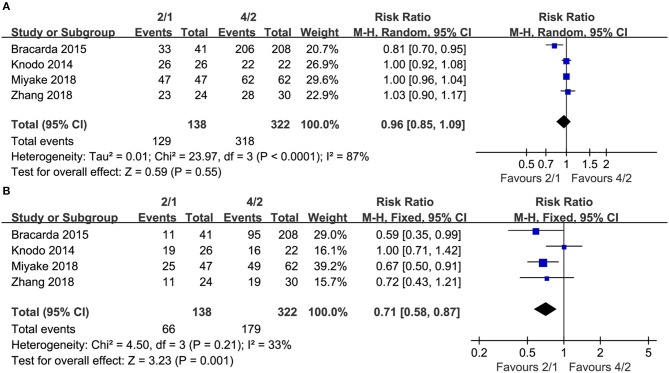
Forest plots of RR of any grade AEs **(A)**, grade 3-4AEs **(B)** associated with 2/1 vs. 4/2.

Four studies compared AEs of any grade (heterogeneity: *I*^2^ = 87%, *P* < 0.0001). No significant differences existed (RR = 0.96, 95% CI: 0.85–1.09, *P* = 0.55; Figure 4A).

Four articles compared grade 3–4 AEs (heterogeneity: *I*^2^ = 33%, *P* = 0.21). No significant differences existed (RR = 0.71, 95% CI: 0.58–0.87, *P* = 0.001; Figure 4B).

**Figure 5:**
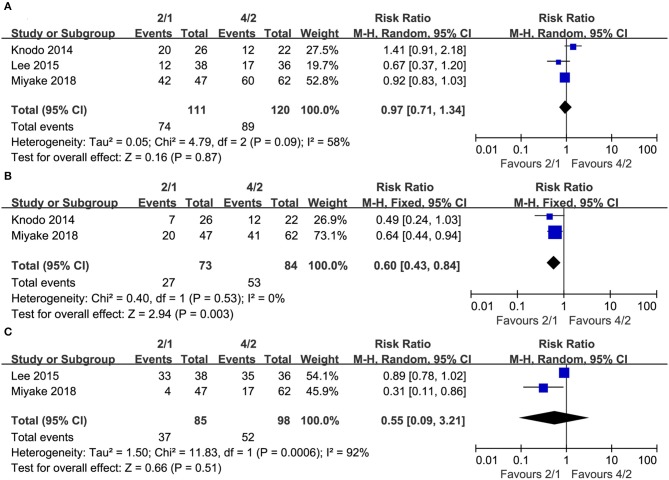
Forest plots of drug reductions **(A)**, drug interruptions **(B)**, and drug discontinuations **(C)** associated with 2/1 vs. 4/2.

Some mRCC patients experienced dose reductions, dose interruptions or dose discontinuations during their treatment. Three studies compared dose reductions (heterogeneity: *I*^2^ = 58%, *P* = 0.09), and no significant differences existed between the two schedules (RR = 0.97, 95% CI: 0.71–1.34, *P* = 0.87; Figure 5A). Two studies compared dose interruptions (heterogeneity: *I*^2^= 0%, *P* = 0.53), and the 2/1 group had fewer dose interruptions than the 4/2 group (RR = 0.60, 95% CI: 0.43–0.84, *P* = 0.003; Figure 5B). Additionally, two articles compared dose discontinuations (heterogeneity: *I*^2^ = 92%, *P* = 0.0006), and no significant differences existed (RR = 0.55, 95% CI: 0.09–3.21, *P* = 0.51; Figure 5C).

In the subanalysis of the ten most common AEs (in order of incidence: leukopenia, thrombocytopenia/platelet disorder, hand-foot syndrome, neutropenia, anemia, hypothyroidism, stomatitis/mucositis, hypertension, fatigue, and abdominal pain/diarrhea), the outcomes of AEs of any grade demonstrated that there was no significant difference in the rates of leukopenia, thrombocytopenia/platelet disorder, and hypothyroidism. Regarding any grade AEs, the 4/2 group had higher incidences of hand-foot syndrome (RR = 0.70, 95% CI: 0.60–0.82, *P* < 0.0001), neutropenia (RR = 0.62, 95% CI: 0.49–0.79, *P* < 0.0001), anemia (RR = 0.80, 95% CI: 0.67–0.95, *P* = 0.01), stomatitis/mucositis (RR = 0.67, 95% CI: 0.54–0.83, *P* = 0.0003), hypertension (RR = 0.65, 95% CI: 0.53–0.79, *P* < 0.0001), fatigue (RR = 0.67,95% CI: 0.59–0.77, *P* < 0.00001), and abdominal pain/diarrhea (RR = 0.67, 95% CI: 0.48–0.92, *P* = 0.02; Table 2) than 2/1. The outcomes of grade 3–4 AEs demonstrated that no significant differences were found for leukopenia, neutropenia, anemia, hypothyroidism, stomatitis/mucositis, or abdominal pain/diarrhea between the two schedules. Within grade 3–4 AEs, the 4/2 schedule had a higher instance of thrombocytopenia/platelet disorder (RR = 0.53, 95% CI: 0.29–0.98, *P* = 0.04), hand-foot syndrome (RR = 0.61, 95% CI: 0.38–0.98, *P* = 0.04), hypertension (RR = 0.45, 95% CI: 0.26–0.77, *P* = 0.004), and fatigue (RR = 0.42, 95% CI: 0.24–0.73, *P* = 0.002; Table 3) than 2/1.

**Table 2:** Top 10 adverse effects (all grade) associated with 2/1 vs. 4/2.

**Adverse effects**	**No. of studies**	**2/1 group (event/total)**	**4/2 group (event/total)**	**RR (95% CI)**	***P*-value**	**Heterogeneity**	
						***I^**2**^* (%)**	***P*-value**
Leukopenia	4	86/135	111/135	0.86 [0.73, 1.00]	0.05	14	0.32
Thrombocytopenia/Platelet disorder	7	124/234	224/438	0.86 [0.70, 1.06]	0.15	64	0.01
Hand-foot syndrome	7	111/234	274/438	0.70 [0.60, 0.82]	<0.0001	15	0.32
Neutropenia	5	59/146	106/168	0.62 [0.49, 0.79]	<0.0001	30	0.22
Anemia	6	92/193	129/230	0.80 [0.67, 0.95]	0.01	40	0.14
Hypothyroidism	6	89/208	188/408	0.83 [0.69, 1.01]	0.06	5	0.39
Stomatitis/Mucositis	5	67/161	205/346	0.67 [0.54, 0.83]	0.0003	0	0.44
Hypertension	7	88/234	227/441	0.65 [0.53, 0.79]	<0.0001	32	0.18
Fatigue	7	117/234	327/438	0.67 [0.59, 0.77]	<0.00001	42	0.11
Abdominal pain/Diarrhea	7	85/234	218/438	0.67 [0.48, 0.92]	0.02	61	0.02

*4/2, 4-weeks-on and 2-weeks-off; 2/1, 2-weeks-on and 1-week-off; RR, risk ratio; CI, confidence interval*.

**Table 3:** Top 10 adverse effects (grade 3–4) associated with 2/1 vs. 4/2.

**Adverse effects**	**No. of studies**	**2/1 group (event/total)**	**4/2 group (event/total)**	**RR (95% CI)**	***P*-value**	**Heterogeneity**	
						***I^**2**^* (%)**	***P*-value**
Leukopenia	4	12/135	18/150	0.76 [0.37, 1.57]	0.46	34	0.21
Thrombocytopenia/Platelet disorder	8	13/255	41/448	0.53 [0.29, 0.98]	0.04	0	0.72
Hand-foot syndrome	8	21/255	56/451	0.61 [0.38, 0.98]	0.04	16	0.31
Neutropenia	7	19/191	33/208	0.62 [0.37, 1.04]	0.07	0	0.46
Anemia	6	15/190	16/210	1.02 [0.52, 1.98]	0.96	0	0.67
Hypothyroidism	6	2/208	9/411	0.58 [0.17, 1.95]	0.38	0	0.67
Stomatitis/Mucositis	5	4/161	23/349	0.38 [0.13, 1.11]	0.08	0	0.74
Hypertension	8	15/255	53/451	0.45 [0.26, 0.77]	0.004	0	0.71
Fatigue	8	14/255	57/451	0.42 [0.24, 0.73]	0.002	0	0.88
Abdominal pain/Diarrhea	6	8/208	24/408	0.75 [0.39, 1.43]	0.38	49	0.08

*4/2, 4-weeks-on and 2-weeks-off; 2/1, 2-weeks-on and 1-week-off; RR, risk ratio; CI, confidence interval*.

### Subgroup Analysis

To determine whether the antitumor effectiveness of the 2/1 and 4/2 schedules were different, we calculated the pooled outcomes of PFS, OS, and ORR in accordance with nationality, treatment line, initial dosage, study quality, and study design (Table 4). Intriguingly, the pooled results of PFS found that the 2/1 schedule had longer PFS (HR = 0.75, 95% CI: 0.58–0.98, *P* = 0.03) among East Asians than other mRCC patients on the same schedule and superior PFS (HR = 0.76, 95% CI: 0.59–0.97, *P* = 0.03) among participants who used an initial dosage of 50 mg/d. Other results of our subanalysis were all robust.

**Table 4:** Subgroup analysis for progression-free survival, overall survival, and objective response rate.

**Group**	**PFS**				**OS**				**ORR**			
	**No. of studies**	**HR (95% CI)**	***P***	***I^**2**^* (%)**	**No. of studies**	**HR (95% CI)**	***P***	***I^**2**^* (%)**	**No. of studies**	**RR (95% CI)**	***P***	***I^**2**^* (%)**
Total	8	0.81 [0.66, 0.99]	0.04	0	6	1.00 [0.95, 1.05]	0.97	0	5	0.91 [0.64, 1.29]	0.58	20
**Nation**												
East Asia	5	0.75 [0.58, 0.98]	0.03	20	3	1.02 [0.69, 1.50]	0.93	0	4	0.92 [0.63, 1.34]	0.65	39
Egypt	1	0.96 [0.51, 1.82]	0.90	NA	1	1.00 [0.87, 1.15]	1	NA	1	0.83 [0.30, 2.31]	0.73	NA
Canada	1	0.84 [0.41, 1.74]	0.64	NA	1	0.63 [0.26, 1.50]	0.3	NA	NA	NA	NA	NA
Italy	1	0.89 [0.57, 1.40]	0.61	NA	1	1.09 [0.44, 2.68]	0.85	NA	NA	NA	NA	NA
**Treatment line**												
First line	4	0.76 [0.58, 1.00]	0.05	0.37	3	0.96 [0.61, 1.51]	0.87	0	2	0.57 [0.31, 1.02]	0.06	0
First and second line	1	0.84 [0.41, 1.74]	0.64	NA	1	0.63 [0.26, 1.50]	0.30	NA	NA	NA	NA	NA
Unclear	3	0.87 [0.63, 1.22]	0.43	0	2	1.00 [0.95, 1.05]	0.97	0	3	1.20 [0.77, 1.89]	0.42	0
**Initial dosage**												
50 mg/d	5	0.76 [0.59, 0.97]	0.03	0	4	1.00 [0.95, 1.05]	0.99	0	3	1.20 [0.77, 1.89]	0.42	0
50/37.5/25 mg/d	1	0.54 [0.21, 1.38]	0.20	NA	NA	NA	NA	NA	1	0.62 [0.30, 1.25]	0.18	NA
Unclear	2	0.99 [0.68, 1.43]	0.94	0	2	0.93 [0.54, 1.63]	0.81	0	1	0.50 [0.18, 1.40]	0.19	NA
**Study quality^a^**												
High quality	5	0.80 [0.64, 1.01]	0.06	16	4	1.03 [0.72, 1.47]	0.87	0	3	1.05 [0.67, 1.64]	0.83	35
Medium quality	3	0.81 [0.53, 1.25]	0.35	0	2	1.00 [0.95, 1.05]	0.95	7	2	0.69 [0.38, 1.24]	0.21	0
**Study design**												
RCT	1	0.75 [0.44, 1.27]	0.29	NA	1	1.15 [0.64, 2.05]	0.64	NA	1	1.42 [0.80, 2.51]	0.23	NA
RS	7	0.82 [0.65, 1.02]	0.07	0	5	1.00 [0.95, 1.05]	0.94	0	4	0.71 [0.45, 1.12]	0.14	0

*PFS, progression-free survival; OS, overall survival; ORR, objective response rate; HR, hazard ratio; RR, risk ratio; CI, confidence interval; NA, not available*.

a *Study quality was evaluated using the Newcastle-Ottawa Scale for retrospective observational studies and the Jadad scale for randomized controlled trials*.

### Sensitivity Analysis

PFS (Figure S1A) and OS (Figure S1B) were both robust, with no estimated value exceeding the 95% CIs. Moreover, the sensitivity analysis of the ORR (Figure S2A) and DCR (Figure S2B) also suggested that there were both consistent outcomes.

### Publication Bias

We did not find any proof of publication bias when analyzing PFS (Begg's test, *P* = 0.711, Egger's test*, P* = 0.656; Figure S3A), OS (Begg's test*, P* = 0.452; Egger's test*, P* = 0.583; Figure S3B), ORR (Begg's test*, P* = 1.000; Egger's test*, P* = 0.360; Figure S4A), or DCR (Begg's test*, P* = 0.734; Egger's test*, P* = 0.528; Figure S4B).

## Discussion

Admittedly, the traditional schedule (4/2) of sunitinib has been associated with some unsatisfactory outcomes, especially severe toxicity, in some mRCC patients. As an alternative, the 2/1 schedule may tackle this dilemma and provide some substantial benefits for mRCC patients. This was the first meta-analysis comparing the effectiveness and toxicity of 2/1 vs. 4/2 sunitinib dosing schedules among patients with mRCC. Our pooled results of nine included studies demonstrated that there was no significant difference in OS and ORR, but the 2/1 schedule was associated with longer PFS, better DCR and fewer drug interruptions. In addition, we found a lower incidence of severe AEs, including thrombocytopenia/platelet disorder, hand-foot syndrome, hypertension, and fatigue, in the 2/1 group than in the 4/2 group. In our subanalysis, the pooled outcomes of studies from East Asian patients reported that the 2/1 schedule was associated with better PFS compared with the same schedule in other mRCC patients, and the 2/1 schedule had also superior PFS among patients who used the initial dosage of 50 mg/d than those administered another initial dosage.

Survival is the most critical point that we should take into account when comparing the 2/1 and 4/2 groups. The pooled outcomes demonstrated that there was no significant difference in OS between the 2/1 and 4/2 schedules, but the 2/1 schedule had an association with improved PFS. In fact, a multicenter phase II RCT suggested that the 2/1 sunitinib dosing schedule had a better failure-free survival rate at half a year than the traditional 4/2 schedule (13). According to a recent RS including 108 Chinese participants, Pan et al. reported that therapy with sunitinib 50 mg/d using a 2/1 schedule could offer better PFS among mRCC patients than the standard schedule 4/2 (19). Additionally, Atkinson et al. demonstrated that among mRCC patients using sunitinib as the first-line treatment, an alternative schedule of sunitinib had a superior median PFS compared to a traditional schedule (14.5 months vs. 4.3 months, *P* < 0.0001) (14). One probable reason may be as follows: severe toxicity of the 4/2 schedule, which could significantly reduce patients' tolerability, influence patients' living quality and give rise to unnecessary drug reductions, interruptions or discontinuations; all these negative events may weaken the antitumor effectiveness of sunitinib in patients on the 4/2 schedule. Remarkably, our subgroup analysis indicated that East Asian patients with mRCC may experience superior PFS compared with other patients, and the 2/1 schedule had superior PFS among patients using an initial dosage of 50 mg/d. Admittedly, positive findings in our subgroup analysis revealed a trend. These conclusions must be accepted with caution, especially the outcomes of subanalyses, and additional high-impact, good-quality RCTs with larger cohorts will be needed to confirm our conclusions.

The response rate is an indispensable cornerstone worth considering when choosing the best dosing schedule of sunitinib. Our pooled results showed that the 2/1 schedule was associated with an equivalent ORR to the 4/2 schedule but a higher DCR. Due to the inconsistent results, we performed an elaborate analysis of the response rate among patients with mRCC. Though patients in the 4/2 group had comparable CR and PR to the patients in the 2/1 group, the latter had more SD (RR = 1.66, 95% CI: 1.19–2.32, *P* = 0.003), which we also regarded as a status of disease control. In light of RECIST 1.1 (Response Evaluation Criteria in Solid Tumors), SD was defined as either a decrease in the overall size of the baseline cancer lesions by <30 percent of the initial size or an increase <20 percent of the initial size (26). In an RS including 154 Japanese participants, Miyake et al. reported no significant differences in response rates between 2 schedules (27.6% vs. 25.8%, *P* = 0.51) (18). Similarly, Din et al. found that both schedules had comparable ORR (23.8% vs. 28.5%), but the 2/1 schedule was associated with more SD than the 4/2 schedule (66.7% vs. 28.6%, *P* = 0.013) at delayed assessment (20). In a single arm phase II study, Jonasch et al. reported a relatively high rate of SD (31%) among patients with mRCC (27). Therefore, we can conclude that the 2/1 schedule had an equivalent ORR (CR+ PR) but a higher SD, which is a significant benefit for patients with mRCC.

The toxicity of sunitinib is also an essential influencing factor when making decisions about 2/1 or 4/2 dosing schedules. Although the 2/1 schedule was not significantly different in dose reductions and dose discontinuations between both dosing schedules, it had fewer dose interruptions (Figure 5). The incidence rates of any grade AEs were not significantly different, but the 4/2 group was associated with higher rates of grade 3–4 AEs than the 2/1 group (Figure 4). In fact, grade 3–4 AEs were a more crucial index of toxicity than grade 1–2 AEs because the compliance of many patients using sunitinib was reduced when grade 3–4 AEs appeared. For grade 3–4 AEs, lower incidences of thrombocytopenia/platelet disorder, hand-foot syndrome, hypertension, and fatigue were reported in the 2/1group. Undoubtedly, our findings demonstrated that sunitinib-treated patients using the 2/1 schedule had fewer sunitinib-related severe AEs and superior tolerability than those using the 4/2 schedule. In an RS analyzing sunitinib-treated participants switching from the 4/2 schedule to the 2/1 schedule, Najjar et al. suggested that therapy on the 2/1 schedule had apparently reduced toxicity among patients experiencing AEs ≥ grade3 in the 4/2 group and could prolong treatment duration greatly (28). An RS reviewed mRCC patients who started therapy with sunitinib on the 4/2 schedule and then switched to 2/1 because of severe AEs, and this analysis found that patients on the2/1schedule had higher quality of life and remarkably lower rates of severe AEs (29). Similarly, some recent studies found that compared with the 4/2 schedule, the 2/1 schedule conveyed a superior quality of life and better tolerability, as reflected by large reductions in some specific toxicities (30–32). In addition, Suo et al. showed that the 2/1 group had much lower mean monthly drug costs than the 4/2 schedule (4,394 Canadian dollars vs. 5936Canadian dollars, *P* < 0.03) (21). Compared with the 4/2 schedule, the 2/1 schedule of sunitinib was the superior dosing schedule for treating mRCC, which balanced toxicity and survival due to fewer sunitinib-related severe AEs, superior PFS and more SD among patients with mRCC.

There were some included studies reporting some sunitinib-treated participants who started treatment using the 4/2 schedule but changed to the 2/1 schedule, and we did not include these patients as either intervention or control groups. There were two main reasons for this. First, the reasons why patients switched from the 4/2 to the 2/1 schedule were varied but may have been due to severe toxicity or disease progression. Second, the time that patients changed from 4/2 to the 2/1 schedule differed, as some patients changed during the first cycle of sunitinib, but other patients changed during later cycles. In brief, the heterogeneity of patients changing from the 4/2 to the 2/1 schedule may be significant, so we believed that it was inappropriate if we included these patients as intervention or control groups.

Some limitations should be taken into account regarding our outcomes. First, the limited number of RCTs (only one) may weaken the quality of these analyses. Second, the number of participants on the two schedules was not large, and this may have resulted in some unreliable estimated values. Third, language bias may exist because all included articles were published in English. Fourth, some outcomes (any grade AEs, dose reductions) had significant heterogeneity, and although they were not the primary index, this might influence the reliability of our conclusions. Fifth, our major outcomes were all low or very low according to the GRADE scale. Sixth, we could not completely control for confounding factors (previous therapy, the number of metastases) because information regarding these factors was sometimes unavailable, but they may have influenced the final results.

## Conclusion

Our meta-analysis demonstrates that the 2/1 schedule has more antitumor benefits (improved PFS, better DCR) than the 4/2 schedule for treating mRCC. Moreover, the 2/1 schedule has less sunitinib-related severe toxicity and better tolerability among patients with mRCC. A 2/1 schedule might produce better PFS among East Asian mRCC patients than in other mRCC patients. In addition, patients administered an initial dosage of 50 mg/d on a 2/1 schedule may have superior PFS. Nevertheless, the inherent limitations of this meta-analysis suggest that more large-scale high-quality studies are required for better determining the role of sunitinib dose schedules under specific clinical circumstances.

## Author Contributions

HD had full access to all of the data in the manuscript and takes responsibility for the integrity of the data and the accuracy of the data analysis. HD and WZ: drafting of the manuscript. HD, ML, QW, LW, ZH, and FY: critical revision of the manuscript for important intellectual content. HD, ML, QW, and LW: statistical analysis. WZ and YW: supervision. All authors: concept and design and acquisition, analysis, or interpretation of data.

### Conflict of Interest

The authors declare that the research was conducted in the absence of any commercial or financial relationships that could be construed as a potential conflict of interest.

## Supplementary Material

Figure S1 Sensitivity analysis of PFS **(A)** and OS **(B)**.

Figure S2 Sensitivity analysis of ORR **(A)** and DCR **(B)**.

Figure S3 Begg's and Egger's tests for comparisons of PFS **(A)** and OS **(B)** associated with 2/1 vs. 4/2.

Figure S4 Begg's and Egger's tests for comparisons of ORR **(A)** and DCR **(B)** associated with 2/1 vs. 4/2.

Table S1 PRISMA 2009 Checklist.

Table S2 Search strategy.

Table S3 Quality assessment of all included studies.

Table S4 GRADE Quality assessment by therapeutic strategy and study design for the outcomes of survival, response rates, and toxicity.

## Abbreviations

4/2m: 4-weeks-on and 2-weeks-off
2/1: 2-weeks-on and 1-week-off
mRCC: metastatic renal cell carcinoma
TKI: tyrosine kinase inhibitor
AEs: adverse effects
HRs: hazard ratios
CR: complete response rate
PR: partial response rate
SD: stable disease rate
ORR: objective response rate
DCR: disease control rate
OS: overall survival
PFS: progression-free survival
RCT: randomized controlled trial
RS: retrospective study
RRs: risk ratios
CIs: confidence intervals
GRADE, Grades of Recommendations Assessment: Development and Evaluation
RECIST: Response Evaluation Criteria in Solid Tumors.
