# Supplementary figures and images for: A 2/1 Sunitinib Dosing Schedule Provides Superior Antitumor Effectiveness and Less Toxicity Than a 4/2 Schedule for Metastatic Renal Cell Carcinoma: A Systematic Review and Meta-Analysis
Source: Front Oncol. 2020 Mar 6;10:313. doi: 10.3389/fonc.2020.00313 (PMC7069552; doi:10.3389/fonc.2020.00313)

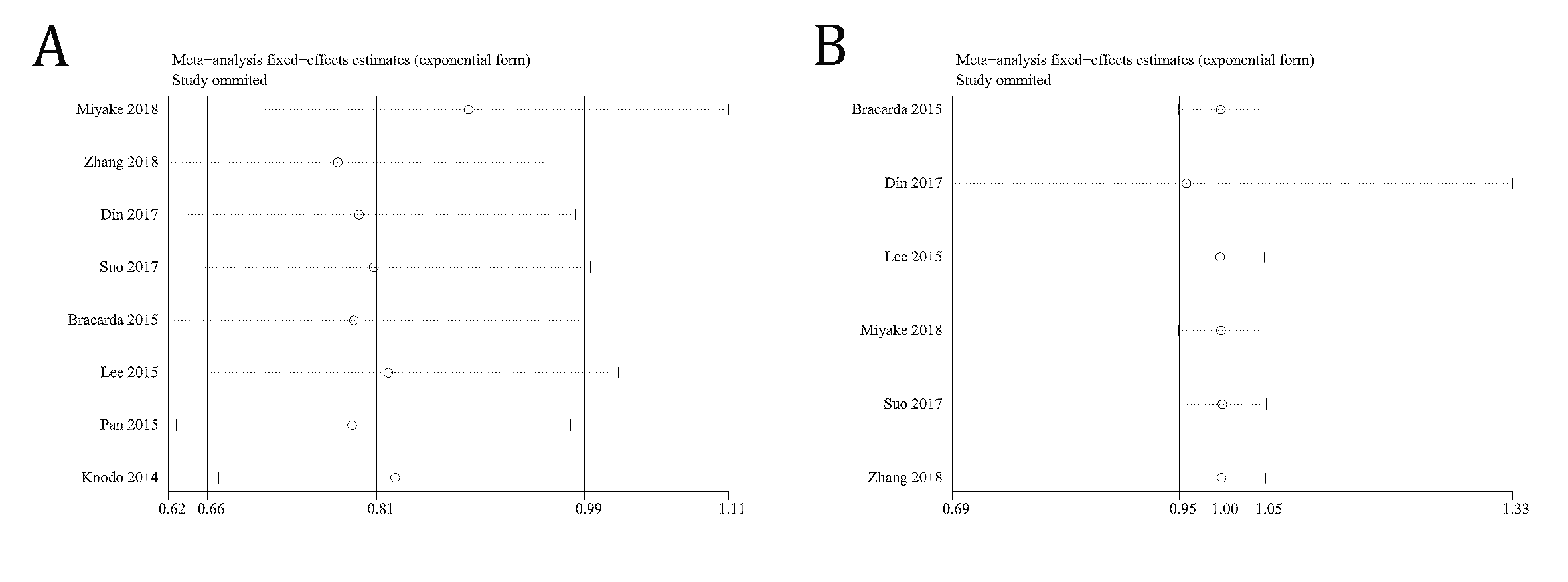

Supplement: Figure S1 — Sensitivity analysis of PFS (A) and OS (B). [file Image_1.TIF]

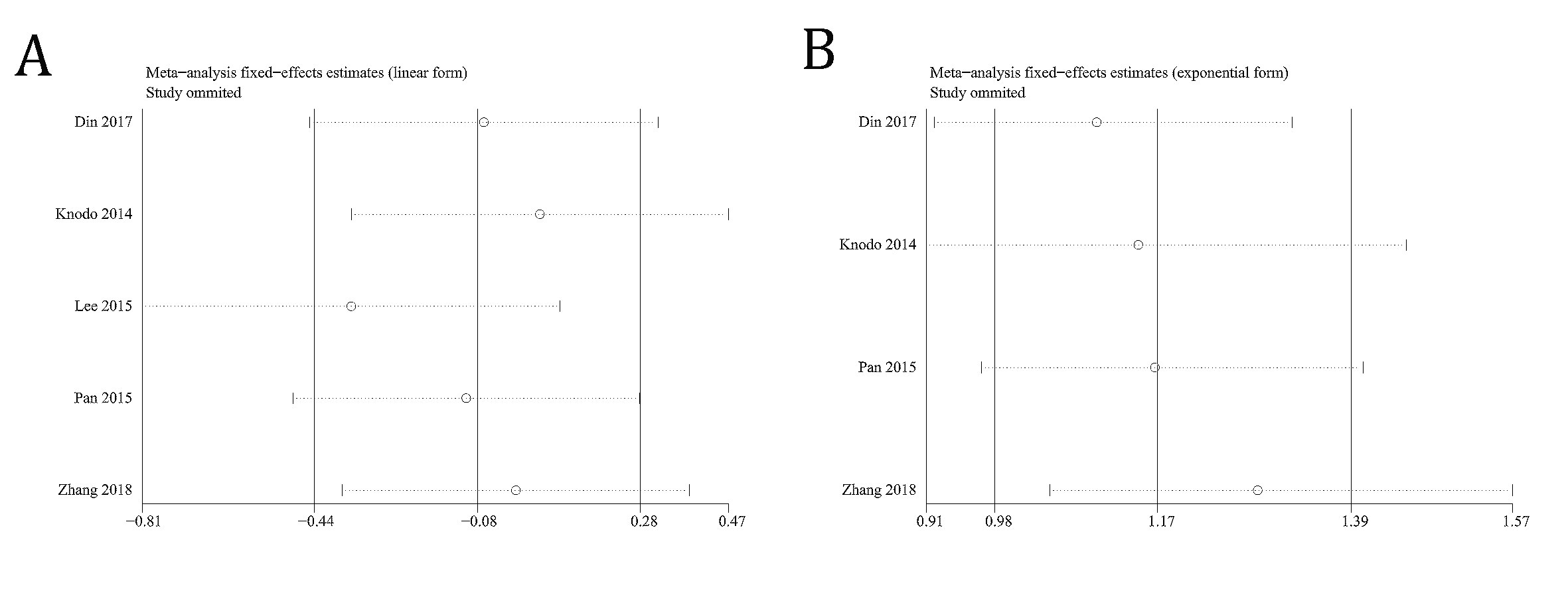

Supplement: Figure S2 — Sensitivity analysis of ORR (A) and DCR (B). [file Image_2.TIF]

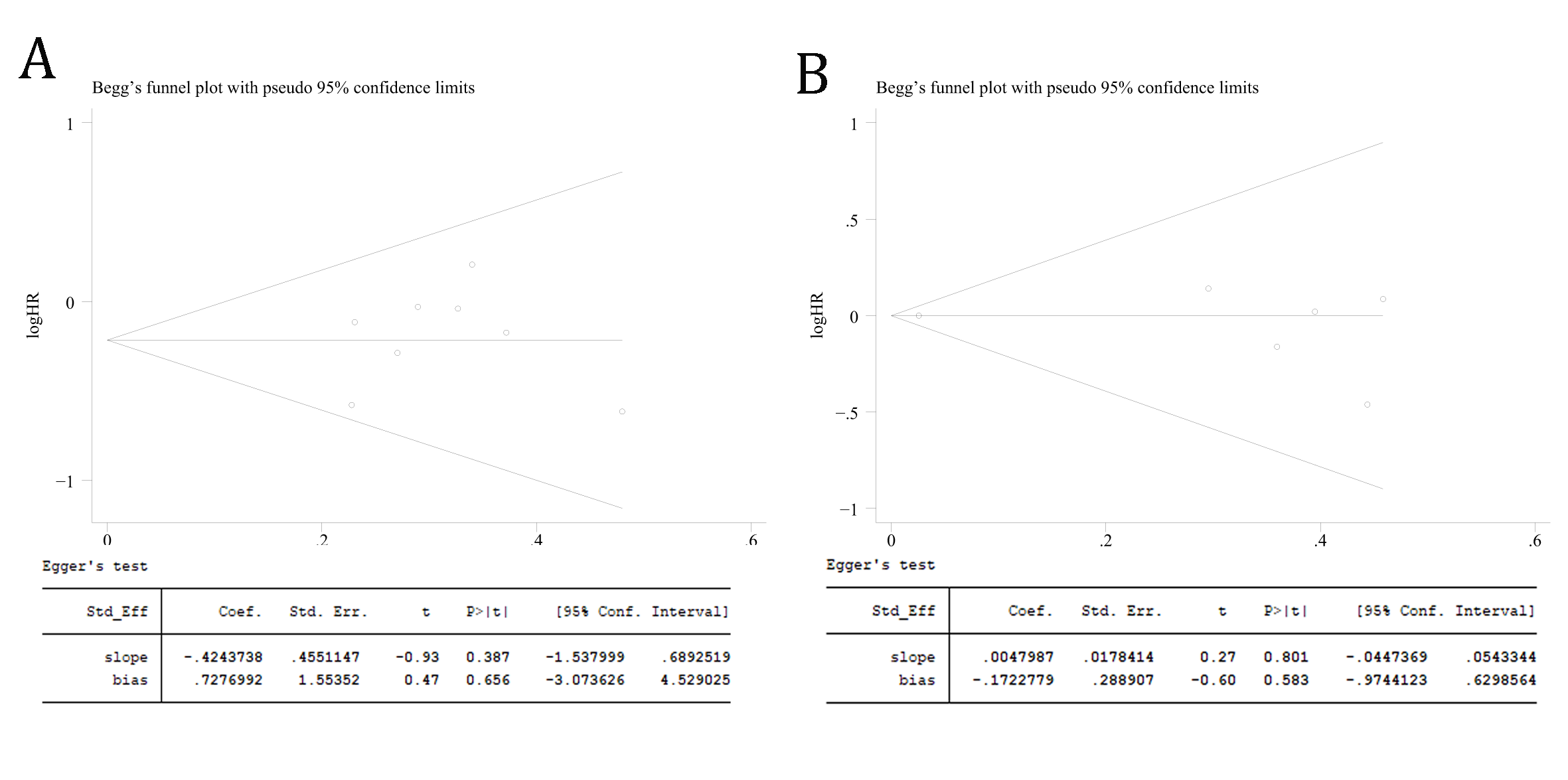

Supplement: Figure S3 — Begg's and Egger's tests for comparisons of PFS (A) and OS (B) associated with 2/1 vs. 4/2. [file Image_3.TIF]

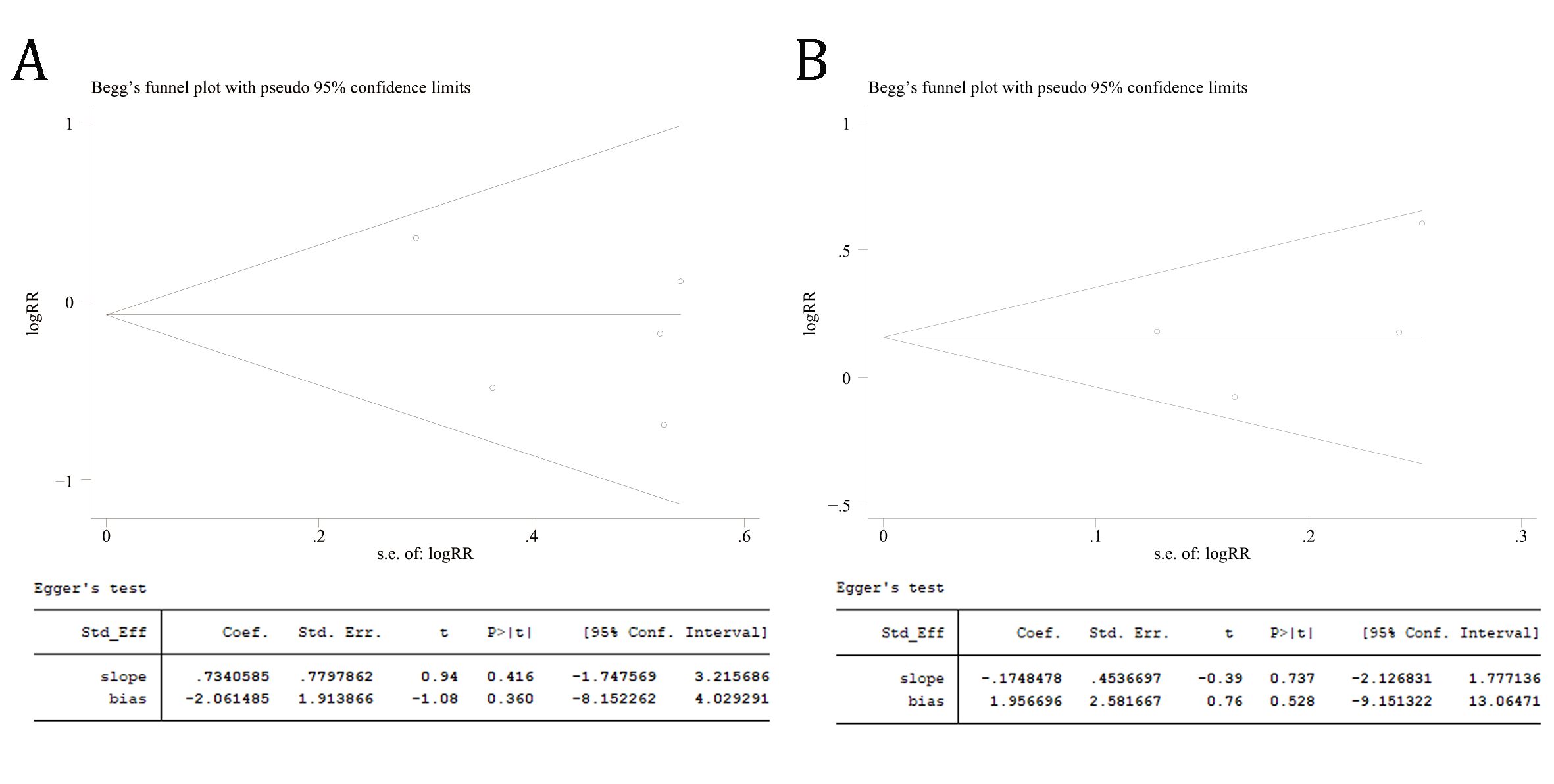

Supplement: Figure S4 — Begg's and Egger's tests for comparisons of ORR (A) and DCR (B) associated with 2/1 vs. 4/2. [file Image_4.TIF]
