# Supplementary material for: A 2/1 Sunitinib Dosing Schedule Provides Superior Antitumor Effectiveness and Less Toxicity Than a 4/2 Schedule for Metastatic Renal Cell Carcinoma: A Systematic Review and Meta-Analysis
Source: Front Oncol. 2020 Mar 6;10:313. doi: 10.3389/fonc.2020.00313 (PMC7069552; doi:10.3389/fonc.2020.00313)
Supplement: Table S2 — Search strategy. [file Table_2.DOCX]

**Appendix 1. Search strategy**

The combined text and medical subject heading (MeSH) terms used were: “Kidney Neoplasm” and “sunitinib”.

**PubMed**

The database was searched on August 5, 2019, n=107.

Search Strategy:

(Kidney Neoplasm [MeSH term] OR Kidney Neoplasm [Text Word] OR Neoplasm, Kidney [Text Word] OR Neoplasm, Renal [Text Word] OR Renal Neoplasm [Text Word] OR Cancer of Kidney [Text Word] OR Renal Cancer [Text Word] OR Kidney Cancer [Text Word] OR Renal Cell Carcinoma [Text Word] OR Renal Cell Cancer [Text Word] OR Clear Cell Renal Cell Carcinoma [Text Word] OR Carcinoma, Renal Cell [Text Word]) AND (Sunitinib [Mesh term] OR sunitinib [Text Word] OR sunitinib malate [Text Word] OR Sutent [Text Word] OR SU 11248 [Text Word] OR SU 011248 [Text Word]) AND (alternative dosing schedule [Text Word] OR 2-weeks-on and 1-week-off [Text Word] OR 4-weeks-on and 2-weeks-off [Text Word] OR two weeks on and one week off [Text Word] OR four weeks on and two weeks off [Text Word] OR Schedule 2/1 [Text Word] OR Schedule 4/2 [Text Word] OR 4/2 Schedule [Text Word] OR 2/1 Schedule [Text Word]).

**Web of Science**

The database was searched on August 5, 2019, n=101.

Search Strategy:

1 TOPIC: (“Kidney Neoplasm” OR “Neoplasm, Kidney” OR “Neoplasm, Renal” OR “Renal Neoplasm” OR “Cancer of Kidney” OR “Renal Cancer” OR “Kidney Cancer” OR “Renal Cell Carcinoma” OR “Renal Cell cancer” OR “Clear Cell Renal Cell Carcinoma” OR “Carcinoma, Renal Cell”) (54013)

2 TOPIC: ("sunitinib" OR "sunitinib malate" OR "Sutent" OR "SU 11248" OR "SU 011248") (8524)

3 TOPIC: ("alternative dosing schedule" OR "2-weeks on/1-week off" OR "4-weeks on/2-weeks off" OR "two weeks on and one week off" OR "four weeks on and two weeks off" OR "Schedule 4/2" OR "Schedule 2/1" OR "4/2 Schedule" OR "2/1 Schedule") (241)

4 #1 AND #2 AND #3 (101)

**EMBASE**

The database was searched on January 5, 2019, n=227.

Search Strategy:

('Kidney Neoplasm':ti,ab,kw OR 'Neoplasm, Kidney':ti,ab,kw OR 'Neoplasm, Renal':ti,ab,kw OR 'Renal Neoplasm':ti,ab,kw OR 'Cancer of Kidney':ti,ab,kw OR 'Renal Cancer':ti,ab,kw OR 'Kidney Cancer':ti,ab,kw OR 'Renal Cell Carcinoma ':ti,ab,kw OR 'Renal Cell Cancer':ti,ab,kw OR 'Clear Cell Renal Cell Carcinoma ':ti,ab,kw OR 'Carcinoma, Renal Cell':ti,ab,kw) AND ('sunitinib':ti,ab,kw OR 'sunitinib malate':ti,ab,kw OR 'Sutent':ti,ab,kw OR 'SU 11248':ti,ab,kw OR 'SU 011248':ti,ab,kw) AND ('alternative dosing schedule':ti,ab,kw OR '2-weeks on/1-week off ':ti,ab,kw OR '4-weeks on/2-weeks off ':ti,ab,kw OR 'two weeks on and one week off ':ti,ab,kw OR 'four weeks on and two weeks off':ti,ab,kw OR 'Schedule 4/2':ti,ab,kw OR ' Schedule 2/1':ti,ab,kw OR '4/2 Schedule':ti,ab,kw OR '2/1 Schedule ':ti,ab,kw)

**Cochrane Library**

The database was searched on August 5, 2019, n=57.

Search Strategy:

(“Kidney Neoplasm” OR “Neoplasm, Kidney” OR “Neoplasm, Renal” OR “Renal Neoplasm” OR “Cancer of Kidney” OR “Renal Cancer” OR “Kidney Cancer” OR “Renal Cell Carcinoma” OR “Renal Cell cancer” OR “Clear Cell Renal Cell Carcinoma” OR “Carcinoma, Renal Cell”): ti,ab,kw AND ("sunitinib" OR "sunitinib malate" OR "Sutent" OR "SU 11248" OR "SU 011248"): ti,ab,kw AND ("alternative dosing schedule" OR "2-weeks on/1-week off" OR "4-weeks on/2-weeks off" OR "two weeks on and one week off" OR "four weeks on and two weeks off" OR "Schedule 4/2" OR "Schedule 2/1" OR "4/2 Schedule" OR "2/1 Schedule"): ti,ab,kw - (Word variations have been searched)

**Ovid MEDLINE**

The database was searched on August 5, 2019, n=65.

Search Strategy:

1 Kidney Neoplasm (6482)

2 Neoplasm, Kidney (17283)

3 Neoplasm, Renal (15008)

4 Renal Neoplasm (609)

5 Cancer of Kidney (1602)

6 Renal Cancer(2286)

7 Kidney Cancer(2386)

8 Renal Cell Carcinoma(10642)

9 Renal Cell cancer (2027)

10 Clear Cell Renal Cell Carcinoma (2350)

11 Carcinoma, Renal Cell(12412)

12 or/1-11 [kidney neoplasm] (17562)

13 sunitinib (4160)

14 sunitinib malate (376)

15 Sutent (214)

16 SU 11248 (22)

17 SU 011248 (2)

18 or/13-17 [sunitinib] (4198)

19 alternative dosing schedule (1289)

20 2-weeks on/1-week off (1249)

21 4-weeks on/2-weeks off (1694)

22 two weeks on and one week off (1282)

23 four weeks on and two weeks off (1539)

24 Schedule 4/2 (2397)

25 Schedule 2/1 (1766)

26 4/2 Schedule (1276)

27 2/1 Schedule (2041)

28 or/19-27 [alternative dosing schedule] (2433)

29 12 and 18 and 28(69)

30 limit 29 to humans (65)

**ScienceDirect**

The database was searched on August 5, 2019, n=104.

Search Strategy:

Title, abstract, keywords: ((“Kidney Neoplasm” OR “Neoplasm, Kidney” OR “Neoplasm, Renal” OR “Renal Neoplasm” OR “Cancer of Kidney” OR “Renal Cancer” OR “Kidney Cancer” OR “Renal Cell Carcinoma” OR “Renal Cell cancer” OR “Clear Cell Renal Cell Carcinoma” OR “Carcinoma, Renal Cell”) and (“sunitinib” OR “sunitinib malate” OR “Sutent” OR “SU 11248” OR “SU 011248”) and ("alternative dosing schedule" OR "2-weeks on/1-week off" OR "4-weeks on/2-weeks off" OR "two weeks on and one week off" OR "four weeks on and two weeks off" OR "Schedule 4/2" OR "Schedule 2/1" OR "4/2 Schedule" OR "2/1 Schedule"))

**Scopus**

The database was searched on August 5, 2019, n=113.

Search Strategy:

TITLE-ABS-KEY ((“Kidney Neoplasm” OR “Neoplasm, Kidney” OR “Neoplasm, Renal” OR “Renal Neoplasm” OR “Cancer of Kidney” OR “Renal Cancer” OR “Kidney Cancer” OR “Renal Cell Carcinoma” OR “Renal Cell cancer” OR “Clear Cell Renal Cell Carcinoma” OR “Carcinoma, Renal Cell”) and (“sunitinib” OR “sunitinib malate” OR “Sutent” OR “SU 11248” OR “SU 011248”) and ("alternative dosing schedule" OR "2-weeks on/1-week off" OR "4-weeks on/2-weeks off" OR "two weeks on and one week off" OR "four weeks on and two weeks off" OR "Schedule 4/2" OR "Schedule 2/1" OR "4/2 Schedule" OR "2/1 Schedule"))
