# Supplementary material for: A 2/1 Sunitinib Dosing Schedule Provides Superior Antitumor Effectiveness and Less Toxicity Than a 4/2 Schedule for Metastatic Renal Cell Carcinoma: A Systematic Review and Meta-Analysis
Source: Front Oncol. 2020 Mar 6;10:313. doi: 10.3389/fonc.2020.00313 (PMC7069552; doi:10.3389/fonc.2020.00313)
Supplement: Table S3 — Quality assessment of all included studies. [file Table_3.DOCX]

**Table S3** Quality assessment of all included studies.

| **Study** | | **Selection** | **Comparability** | **Exposure** | **Randomization** | **Masking** | **Accountability of all patients** | **Quality (score)** |
| --- | --- | --- | --- | --- | --- | --- | --- | --- |
| **RCT** a | | | | | | | | |
| 2015 | Lee [12] |  |  |  | ★★ | ★ | ★ | 4 |
| **RS** b | | | | | | | | |
| 2018 | Miyake [18] | ★★★ | ★★ | ★★★ |  |  |  | 8 |
| 2015 | Pan [19] | ★★★ | ★★ | ★★★ |  |  |  | 8 |
| 2017 | Din [20] | ★★ | ★★ | ★★ |  |  |  | 6 |
| 2017 | Suo [21] | ★★★ | ★★ | ★★ |  |  |  | 7 |
| 2014 | Knodo [22] | ★★ | ★★ | ★★★ |  |  |  | 7 |
| 2018 | Zhang [23] | ★★★ | ★★ | ★★★ |  |  |  | 8 |
| 2013 | Neri [24] | ★★ | ★★ | ★★★ |  |  |  | 7 |
| 2015 | Bracarda1 [25] | ★★★ | ★★ | ★★★ |  |  |  | 8 |

**Abbreviations:** RCT: randomized controlled trial; RS: retrospective study.

^a^ RCT was assessed using the Jadad scale;

^b^ RS were assessed using the Newcastle-Ottawa Scale.
