# Supplementary material for: A 2/1 Sunitinib Dosing Schedule Provides Superior Antitumor Effectiveness and Less Toxicity Than a 4/2 Schedule for Metastatic Renal Cell Carcinoma: A Systematic Review and Meta-Analysis
Source: Front Oncol. 2020 Mar 6;10:313. doi: 10.3389/fonc.2020.00313 (PMC7069552; doi:10.3389/fonc.2020.00313)
Supplement: Table S4 — GRADE Quality assessment by therapeutic strategy and study design for the outcomes of survival, response rates, and toxicity. [file Table_4.DOCX]

**Table S4** GRADE Quality assessment by therapeutic strategy and study design for the outcomes of survival, response rates, and toxicity.

| **Primary outcomes** | **No. of Studies** | **No. of participants** | | **Differences^a^（95%CI）** | **Quality assessment** | | | | | | **Quality** |
| --- | --- | --- | --- | --- | --- | --- | --- | --- | --- | --- | --- |
|  |  | **2/1** | **4/2** |  | **Risk of bias^b^** | **Inconsistency** | **Indirectness** | **Imprecision** | **Publication bias^c^** | |  |
| **Survival** |  |  |  |  |  |  |  |  | |  |  |
| PFS | 8 | 243 | 500 | 0.81 [0.66, 0.99] | Serious (-1) | No inconsistency | No indirectness | Serious (-1) | | Unlikely | Low |
| OS | 6 | 185 | 428 | 1.00 [0.95, 1.05] | Serious (-1) | No inconsistency | No indirectness | Serious (-1) | | Unlikely | Low |
| **Response rates** |  |  |  |  |  |  |  |  | |  |  |
| ORR | 5 | 40/141 | 46/159 | 0.91 [0.64, 1.29] | Serious (-1) | Serious (-1) | No indirectness | Serious (-1) | | Unlikely | Very Low |
| DCR | 4 | 76/108 | 73/132 | 1.22 [1.01, 1.47] | Unclear | Very Serious (-2) | No indirectness | No imprecision | | Unlikely | Low |
| CR | 5 | 5/141 | 6/159 | 0.93 [0.31, 2.79] | Serious (-1) | No inconsistency | No indirectness | Very Serious (-2) | | Unlikely | Very Low |
| PR | 5 | 35/141 | 40/159 | 0.90 [0.61, 1.33] | Serious (-1) | Serious (-1) | No indirectness | Serious (-1) | | Unlikely | Very Low |
| SD | 4 | 54/108 | 39/132 | 1.66 [1.19, 2.32] | Unclear | Serious (-1) | No indirectness | Serious (-1) | | Unlikely | Low |
| **Toxicity** |  |  |  |  |  |  |  |  | |  |  |
| Any grade AEs | 4 | 129/138 | 318/322 | 0.93 [0.87, 0.99] | Unclear | Very Serious (-2) | No indirectness | No imprecision | | Unlikely | Low |
| Grade 3-4 AEs | 4 | 66/138 | 179/322 | 0.71 [0.58, 0.87] | Serious (-1) | Serious (-1) | No indirectness | No imprecision | | Unlikely | Low |
| Dose reductions | 3 | 74/111 | 89/120 | 0.97 [0.71, 1.34] | Serious (-1) | Very Serious (-2) | No indirectness | No imprecision | | Unlikely | Very Low |
| Dose interruptions | 2 | 27/73 | 53/84 | 0.60 [0.43, 0.84] | Serious (-1) | No inconsistency | No indirectness | Serious (-1) | | Unlikely | Low |
| Dose discontinuations | 2 | 37/85 | 52/98 | 0.55 [0.09, 3.21] | Serious (-1) | Very Serious (-2) | No indirectness | Very Serious (-2) | | Unlikely | Very Low |
| Any grade leukopenia | 4 | 86/135 | 111/150 | 0.86 [0.73, 1.00] | Serious (-1) | Serious (-1) | No indirectness | No imprecision | | Unlikely | Low |
| Grade 3-4 leukopenia | 4 | 12/135 | 18/150 | 0.76 [0.37, 1.57] | Serious (-1) | Serious (-1) | No indirectness | Serious (-1) | | Unlikely | Very Low |
| Any grade thrombocytopenia | 7 | 124/234 | 224/438 | 0.86 [0.70, 1.06] | Serious (-1) | Very Serious (-2) | No indirectness | No imprecision | | Unlikely | Very Low |
| / platelet disorder |  |  |  |  |  |  |  |  |  |  |  |
| Grade 3-4 thrombocytopenia | 8 | 13/255 | 41/448 | 0.53 [0.29, 0.98] | Serious (-1) | No inconsistency | No indirectness | Serious (-1) | | Unlikely | Low |
| /platelet disorder |  |  |  |  |  |  |  |  |  |  |  |
| Any grade hand-foot syndrome | 7 | 111/234 | 274/438 | 0.70 [0.60, 0.82] | Serious (-1) | Serious (-1) | No indirectness | No imprecision | | Unlikely | Low |
| Grade 3-4 hand-foot syndrome | 8 | 21/255 | 56/451 | 0.61 [0.38, 0.98] | Unclear | Serious (-1) | No indirectness | Serious (-1) | | Unlikely | Low |
| Any grade neutropenia | 5 | 59/146 | 106/168 | 0.62 [0.49, 0.79] | Serious (-1) | Serious (-1) | No indirectness | No imprecision | | Unlikely | Low |
| Grade 3-4 neutropenia | 6 | 18/167 | 31/178 | 0.62 [0.36, 1.06] | Serious (-1) | Serious (-1) | No indirectness | Serious (-1) | | Unlikely | Very Low |
| Any grade anemia | 6 | 92/193 | 129/230 | 0.80 [0.67, 0.95] | Serious (-1) | Serious (-1) | No indirectness | No imprecision | | Unlikely | Low |
| Grade 3-4 anemia | 7 | 16/214 | 18/240 | 0.98 [0.52, 1.85] | Serious (-1) | No inconsistency | No indirectness | Serious (-1) | | Unlikely | Low |
| Any grade hypothyroidism | 6 | 89/208 | 188/408 | 0.83 [0.69, 1.01] | Unclear | Serious (-1) | No indirectness | Serious (-1) | | Unlikely | Low |
| Grade 3-4 hypothyroidism | 6 | 2/208 | 9/411 | 0.58 [0.17, 1.95] | Serious (-1) | Serious (-1) | No indirectness | Very Serious (-2) | | Unlikely | Very Low |
| Any grade stomatitis/  mucositis | 5 | 67/161 | 205/346 | 0.67 [0.54, 0.83] | Serious (-1) | Serious (-1) | No indirectness | No imprecision | | Unlikely | Low |
| Grade 3-4 stomatitis/  mucositis | 5 | 4/161 | 23/349 | 0.38 [0.13, 1.11] | Serious (-1) | No inconsistency | No indirectness | Serious (-1) | | Unlikely | Low |
| Any grade hypertension | 7 | 88/234 | 227/441 | 0.65 [0.53, 0.79] | Serious (-1) | Serious (-1) | No indirectness | No imprecision | | Unlikely | Low |
| Grade 3-4 hypertension | 8 | 15/255 | 53/451 | 0.45 [0.26, 0.77] | Serious (-1) | No inconsistency | No indirectness | Serious (-1) | | Unlikely | Low |
| Any grade fatigue | 7 | 117/234 | 327/438 | 0.67 [0.59, 0.77] | Unclear | Very Serious (-2) | No indirectness | Serious (-1) | | Unlikely | Very Low |
| Grade 3-4 fatigue | 8 | 14/255 | 57/451 | 0.42 [0.24, 0.73] | Serious (-1) | No inconsistency | No indirectness | Serious (-1) | | Unlikely | Low |
| Any grade abdominal pain | 7 | 85/234 | 218/438 | 0.71 [0.59, 0.85] | Serious (-1) | Very Serious (-2) | No indirectness | No imprecision | | Unlikely | Very Low |
| /diarrhea |  |  |  |  |  |  |  |  |  |  |  |
| Grade 3-4 abdominal pain | 6 | 8/208 | 24/408 | 0.75 [0.39, 1.43] | Unclear | Very Serious (-2) | No indirectness | Serious (-1) | | Unlikely | Very Low |
| /diarrhea |  |  |  |  |  |  |  |  |  |  |  |

**Abbreviations:** PFS: progression-free survival; OS: overall survival; ORR: objective response rate; DCR: disease controlrate; CR: complete response rate; PR: partial response rate; SD: stable disease rate; 4/2: 4-weeks-on and 2-weeks-off; 2/1: 2-weeks-on and 1-week-off; AEs: adverse effects; CI: confidence interval.

^a^ Differences: hazard ratio (HR) for PFS and OS; risk ratios (RR) for CR; PR; SD; ORR; DCR; any grade AEs; grade 3-4 AEs; dose interruptions; dose reductions;

dose discontinuations; and any grade and grade 3-4 AEs of the top 10 AEs.

^b^ Risk of bias assessed using the Newcastle-Ottawa Scale (NOS) for non-randomized studies and the Jadad scale for randomized controlled trials.

^c^ Publication bias was assessed by Egger’s and Begg’s tests.
